# Gangrene Treated by Turpentine

**Published:** 1871-12

**Authors:** 


Gangrene Treated by Turpentine.—Dr. Lange mentions a case of gangrene in which turpentine, after other remedies had proved ineffectual, not only saved the patient's life, but also nearly all the parts affected. A girl eleven years old had gangrenous destruction of the right cheek. The part was treated with turpentine on lint, and the dressing changed every two hours.—*Indiana Journal of Medicine.*

